# Brain state-dependent neuronal computation

**DOI:** 10.3389/fncom.2012.00077

**Published:** 2012-10-01

**Authors:** Pascale P. Quilichini, Christophe Bernard

**Affiliations:** ^1^Aix Marseille Université, INSMarseille, France; ^2^Inserm, UMR_S 1106Marseille, France

**Keywords:** oscillation, information processing, brain state, resonance, neuromodulator

## Abstract

Neuronal firing pattern, which includes both the frequency and the timing of action potentials, is a key component of information processing in the brain. Although the relationship between neuronal output (the firing pattern) and function (during a task/behavior) is not fully understood, there is now considerable evidence that a given neuron can show very different firing patterns according to brain state. Thus, such neurons assembled into neuronal networks generate different rhythms (e.g., theta, gamma and sharp wave ripples), which sign specific brain states (e.g., learning, sleep). This implies that a given neuronal network, defined by its hard-wired physical connectivity, can support different brain state-dependent activities through the modulation of its functional connectivity. Here, we review data demonstrating that not only the firing pattern, but also the functional connections between neurons, can change dynamically. We then explore the possible mechanisms of such versatility, focusing on the intrinsic properties of neurons and the properties of the synapses they establish, and how they can be modified by neuromodulators, i.e., the different ways that neurons can use to switch from one mode of communication to the other.

Observations obtained during specific behavioral tasks or cognitive functions provide most of our knowledge about information processing in the brain. Electrophysiological recordings during different contexts are characterized by the presence of oscillations in specific frequency bands; and a change in the firing pattern of neurons (Buzsaki, 2006). Both phenomena are intimately linked since oscillations are generated/controlled by the firing of neurons, and neuronal firing pattern is itself directly influenced by the ongoing network oscillation (Buzsaki, 2006). Different brain states are associated with different rhythms, and different brain rhythms are associated with different firing patterns of neurons (Buzsaki, 2006). For example, processing of spatial information is associated with oscillations in the theta band (4–12 Hz), while storage of spatial memory occurs during sharp wave ripples (>100 Hz). During these activities, neurons display different firing patterns (Klausberger et al., 2003; Klausberger and Somogyi, 2008). Understanding neuronal computation as a function of brain state thus requires knowing how neuronal networks can switch from one mode of oscillation to another as well as how neurons can switch from one firing pattern to another. Both questions are part of the same problem and cannot be dissociated.

Brain oscillations are emerging properties of neuronal networks; they depend upon connections and time-delays (Deco et al., 2011). Connections must be understood as everything that covers the way a given source neuron transmits information to its targets. This includes not only the wiring diagram, but also the intrinsic properties of the source neuron as well as the presynaptic and postsynaptic properties. Hence, connection means here functional connectivity. As a working hypothesis, we propose that changes in functional connectivity contribute to brain state-dependent changes in oscillatory modes and firing patterns. This hypothesis is difficult to test because of the diversity of cell types in the brain. Most cortical regions contain a majority of principal glutamatergic cells (80–90%) and GABA neurons (10–20%). Although principal cells appear rather homogeneous, GABA neurons are heterogeneous (Klausberger and Somogyi, 2008). Neurons are connected to each other in a source- and target-dependent manner, some GABA neurons displaying highly specialized and precise connectivity patterns (Klausberger and Somogyi, 2008). Hence, it can be proposed that subsets of neuron types may be engaged in a brain state-dependent manner.

## Oscillations are generated by neurons, and, in turn, oscillations influence their firing

Place cells are exemplifying this concept. The hippocampus is central for encoding spatial information. The activity of CA1 pyramidal cells (both the rate and the timing, or phase, of action potentials relative to the oscillation cycle) is finely modulated by theta rhythm, which is observed during exploration. A given CA1 pyramidal cell can encode for a specific place in the environment. Its firing rate increases as the animal gets closer to the center of the place field, but as interestingly, the spikes of the place cell shift backward relative to the phase of the ongoing theta oscillations (O'Keefe and Recce, 1993). Such phase-precession represents a temporal code, for it causally relates the timing of the principal cells' spikes to the behavior of the animal, hence the oscillatory state.

**Figure 1:**
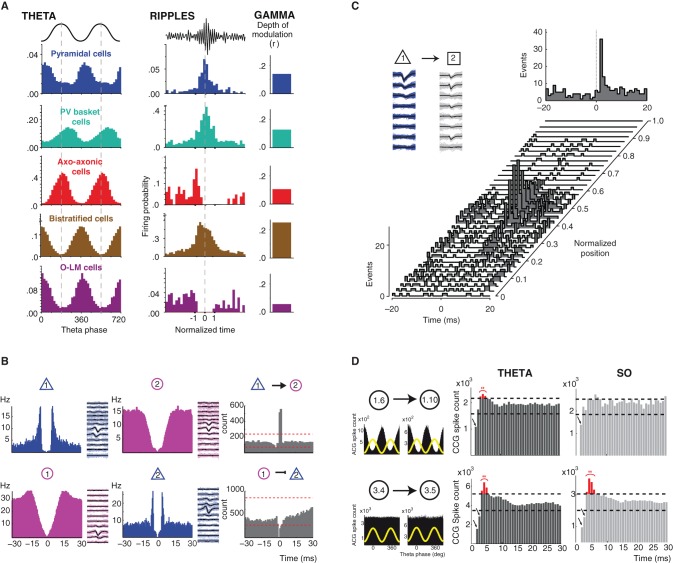
**Brain state/oscillation dependent modulation of neuronal activity and functional connectivity. (A)** Oscillation-dependent firing profiles. Distinct classes of hippocampal GABA neurons display different and specific firing patterns (firing probability histograms) during theta and ripple oscillations (their spike timing is coupled to field gamma oscillations to differing degrees). Modified from (Klausberger and Somogyi, 2008). **(B)** Identification of putative functional connectivity among neurons. Autocorrelograms and average filtered waveforms of a putative principal cell (blue) and an interneuron (purple) in the entorhinal cortex in layer 2 (top panel) and layer 3 (bottom panel). Cross-correlogram (CCG, grey) reveals short-latency monosynaptic excitation between neuron 1 and neuron 2 (top panel) and short-latency suppression of spikes in the target principal neuron (bottom panel). Modified from (Quilichini et al., 2010). **(C)** Behavior-dependent changes in monosynaptic interactions. Short-term cross-correlograms between a putative pyramidal cell (cell 1, mean waveform in black and single spikes in blue) and interneuron (cell 2, mean waveform in black and single spikes in grey) in the medial prefrontal cortex as a function of the rat's position on the central arm of a figure-eight-T-maze before a left turn. A significant functional connection between the cell 1 and 2 is only revealed by the CCGs around the center of the arm. Top right panel, cross-correlograms session mean. Modified from (Fujisawa et al., 2008). **(D)** Modulation of functional connectivity by brain state dependent oscillations. In the entorhinal cortex superficial layers (2 and 3), a portion of pairs between putative interneurons (1.6 presynaptic neuron; 1.10 postsynaptic neuron) displaying a strong theta-phase modulation of their firing (top left panel, theta phase distribution of spikes in black and theta cycle as yellow wave) show a brain state dependent expression of post-inhibitory rebound (PIR) only during theta oscillations (red bins in the CCGs). However, the expression of PIR did not depend upon the oscillatory activity (theta vs. slow oscillations) in theta-phase unmodulated pairs of putative interneurons (3.4 presynaptic neuron; 3.5 postsynaptic neuron; bottom panel). Modified from (Adhikari et al., 2012).

Although GABA neurons do not show much phase precession (Maurer et al., 2006; Ego-Stengel and Wilson, 2007; Geisler et al., 2007), their firing is strongly shaped by the ongoing oscillatory activity, i.e., the brain-state. The work of T. Klausberger and collaborators emphasizes how the output of different hippocampal CA1 GABA neurons is constrained by the ongoing theta, gamma oscillations, and ripples oscillations (Klausberger et al., 2003, 2004, 2005; Klausberger and Somogyi, 2008; Klausberger, 2009). Different types of GABA neurons display different firing patterns during a given oscillation (e.g. parvalbumin-containing basket cells and axo-axonic cells fire out of phase compared to pyramidal neurons during theta) and a given cell type displays different firing patterns during different oscillations (for example, O-LM cells fire during theta, but stop firing during ripple oscillations) (Figure 1A). This mosaic of oscillation-dependent firing profiles endows the brain with a true arsenal to organize the activity of different cells assemblies according to the output function/behavior, enabling different coding/computation modes: for instance encoding of space by place cells during exploration (theta oscillations) and consolidation of such information during slow-wave sleep (sharp-wave ripples complexes) (Moser et al., 2008; Girardeau and Zugaro, 2011). The different firing patterns of GABA neurons during different brain states suggest that neurons may perform different types of computation according to the context. Although we do not fully understand the functional meaning of these different firing patterns, there is evidence that a given neuron can encode different features of the environment.

## One neuron, different codes

The firing activity of a given neuron may contain information based on different coding schemes: the “temporal code,” based on the precise timing of the spikes and the “rate code,” when the information is represented by a modulation of the firing rate (Gerstner et al., 1997). Here also, the example of place cells is also striking. Although one neuron can exhibit a spatially localized firing to describe a place field, the place cell representation can suddenly change its activity from one pattern to another in response to changes in the environment. This phenomenon, known as remapping, is a well-known example of one neuron coding for different information (Muller and Kubie, 1987; Colgin et al., 2008; Jeffery, 2011). When some aspects of the environment are changed, like the shape of the testing enclosure, a proportion of neurons show unrelated place fields in the other environment (Muller and Kubie, 1987). Other factors, like light, color, or sensory changes also produce remapping (Quirk et al., 1990; Bostock et al., 1991; Markus et al., 1995; Moita et al., 2004; Colgin et al., 2008).

There are different patterns of remapping: the remapping of place cell activity can include a substantial change of its firing rate (i.e., “rate remapping”) or a global, even complete change of both firing field and rate (i.e., “global remapping”). Rate remapping and global remapping have been proposed to represent distinct hippocampal encoding systems (Leutgeb et al., 2005). In rate remapping, the population of active cells and the location of the place fields remain unchanged, suggesting that the change in rate represents non-spatial aspects of an experience on top of a stable place code. Global remapping, however, is an all-or-none mechanism, and would code for a more substantial degree of difference in the environment. Such a phenomenon would allow different memories to be separated and stored.

Single cell recordings performed in the human brain (in the context of epilepsy neurosurgery) also revealed multi-information processing. A given neuron can display a sparse but consistent response to different pictures (Bahai Temple, Sydney Opera, and Jeniffer Aniston together with Brad Pitt, but not of snakes) shown to the patient (Quiroga et al., 2005, 2008). These results show that a given neuron can code for very different information. Hence, hippocampal neurons possess a multi-representational nature, which is essential for a structure involved in high-capacity memory storage.

Nevertheless, the sole coding of individual neurons does not appear sufficient to represent detailed descriptions of relevant features of the environment. It is now generally accepted that unambiguous representations are based on population codes. The activity of neuronal populations (or networks) depends upon the way neurons are connected to each other. Interestingly, the connectivity diagram also shows remarkable versatility and dynamic remodeling.

## Functional connections are as versatile as neuronal firing

In the neocortex, neurons are interconnected with each other in a direct (monosynaptic connection) and indirect (polysynaptic interactions) fashion. They form hard-wired networks, where the information can flow within and across layers via the axons. Such pathways represent the anatomical connectivity, describing how local and larger networks are physically distributed and linked. The information travels dynamically through these networks, according to the fluctuation of the ongoing activity. When a neuron emits an action potential, neurotransmitter will be released at presynaptic sites, activating postsynaptic receptors, usually leading to depolarization or hyperpolarization of the target cells. Hence, the functional consequence of the firing of the presynaptic cell will be an increased or decreased probability of firing for the postsynaptic cells. This mode of information transfer between two neurons can be revealed *in vivo* by the cross-correlation function (i.e., cross-correlograms, CCG) between their respective spike trains, which quantifies how much the firing of a one neuron is positively or negatively correlated with the firing of the other neuron within a relatively small time window (Csicsvari et al., 1998; Bartho et al., 2004; Fujisawa et al., 2008; Ostojic et al., 2009; Quilichini et al., 2010; Adhikari et al., 2012) (Figure 1B). CCGs can thus be used to identify putative direct synaptic connections between neurons (Moore et al., 1970). There is now evidence that synaptic connections can be dynamically modulated in a brain state-dependent manner, for example when an animal runs on the central arm of the maze in an alternating task, i.e., choice to turn left or right (Fujisawa et al., 2008) (Figure 1C). Such dynamic modulation of connections between neurons enables a reconfiguration of neuronal assemblies, which output might reflect a neuronal representation of goals and trajectory. This is the first demonstration of a variation of functional connectivity as a function of the task in which the animal is engaged.

In the entorhinal cortex, different brain state dependent oscillations also modulate functional connectivity among neurons (Adhikari et al., 2012). Inhibitory connections and the presence of a post-inhibitory rebound action potential (PIR) between pairs of putative GABA neurons display a brain state preference: their expression being more prominent during theta oscillation as compared to slow oscillations (Figure 1D). These data show how a given network of neurons can functionally reorganize its functional architecture thought different oscillatory states, hence in order to support different output. Such a mechanism might be the result and/or serve to the emergence of oscillations and to achieve global network synchronization and transition between brain states.

Obviously, the ability to express different firing patterns and functional connectivity increases the computational power of neuronal networks. Such functional reconfiguration allows the transient constitution of specific sub networks in a brain state-dependent manner. Thus, neurons can be engaged in different functions. We will now review the mechanisms that may underlie the versatility of firing patterns and connections.

## Underlying mechanisms: cellular (intrinsic properties)

The firing pattern of a neuron depends upon the way synaptic inputs interact with ionic channels. The first step is reaching the threshold for action potential initiation. Once an action potential is generated, others can be triggered, via a combination of multiple mechanisms. A cell can be a natural burster, i.e., once reaching the threshold for action potential initiation; several spikes are emitted because the cell remains depolarized, for example via the activation of persistent sodium channels or calcium channels. The burst pattern depends upon the biophysics of the different ionic channels (for example, recovery from inactivation) and their respective pattern of activation (for example, Ca^2+^-dependent K^+^ channels strongly influence the firing pattern). Since different Na^+^, Ca^2+^, and K^+^ channels can interact to shape neuronal output, and since different types of neurons can express different sets of channels, there are multiple ways to produce different firing patterns based on the sole intrinsic properties. Alternatively, a cell may emit a single action potential despite receiving strong depolarizing synaptic inputs, because of a stronger activation of K^+^ channels, which will prevent the membrane potential to reach the action potential threshold.

**Figure 2:**
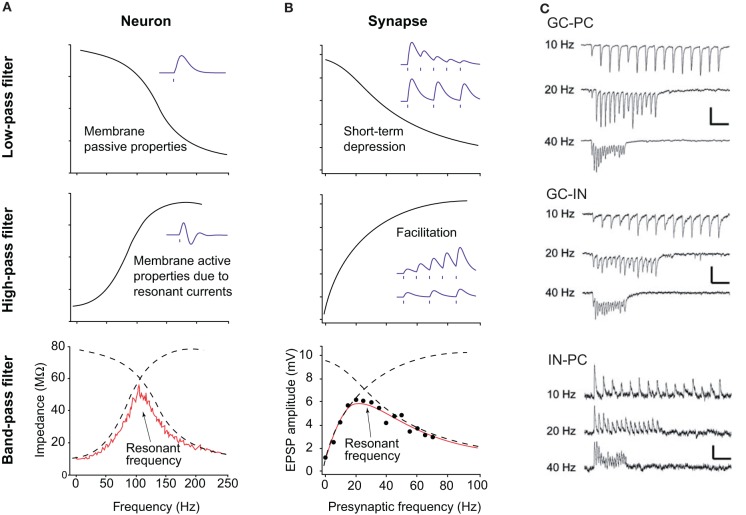
**Principles of resonance and short-term plasticity of synapses. (A)** Resonance in neurons. (Top panel) The capacitive properties of the neuronal membrane act as a low pass filter, efficiently dampening high frequency inputs. (Middle panel) The presence of ionic channels, like I_h_ or I_M_, provide high pass properties. Bottom panel. The combination of low and high pass filters makes a pass band filter, with a resonant frequency, i.e., the frequency favored by the cell. **(B)** Resonance at the synapse. (Top panel) Some synapses, when activated at a given frequency, display short-term depression (i.e., the amplitude of the postsynaptic response decreases), thus making a low pass filter. (Middle panel) Other synapses facilitate (i.e., the amplitude increases), making a high pass filter. The combination of both types of synapses also makes a pass band filter, with an optimal resonance frequency. **(C)** Examples of frequency-dependent short term plasticity. Different connections are tested: dentate granule cell to CA3 pyramidal cell (top), dentate granule cell to CA3 interneuron (middle), CA3 interneuron to pyramidal cell (bottom). Note the switch from facilitation to depression at excitatory synapses between 10 and 40 Hz, and strong depression at 40 Hz at inhibitory connections. Adapted from Izhikevich et al. (2003) and Mori et al. (2004).

Resonance properties provide another important mechanism that constrains the firing pattern of some neurons (Figure 2). As mentioned above, many types of oscillations can be recorded in neuronal circuits, from very slow (<0.1 Hz) to very fast (100–200 Hz). The frequency of the synaptic inputs often reflects the field frequency. However, neurons do not process each input equally. First, the membrane capacitance makes the cell act as a low pass filter. According to the value of capacitance, which varies from one cell type to another, neurons will be more or less sensitive to high frequency inputs (Figure 2A). Second, many cell types possess ionic channels conferring high pass filtering properties (Hutcheon and Yarom, 2000; Izhikevich et al., 2003). Among them, one can distinguish I_h_, I_M_, and I_NaP_. They act as an inductance L. The combination of the capacitive and inductive effect produces a pass band filter (Figure 2A). In CA1 pyramidal cells, I_h_ provides strong resonance properties in the theta frequency range (4–12 Hz), in particular in the dendrites (Narayanan and Johnston, 2007; Marcelin et al., 2009). Such resonance properties may be involved in the theta modulation of place cells.

Interestingly, different levels of I_h_ expression in the dorsal and ventral hippocampus endow with different resonance properties (Marcelin et al., 2012a,b), which may be linked to the different properties of place cells along the dorsal-ventral axis (Kjelstrup et al., 2008). GABA neurons can also express resonance properties, including O-LM cells and basket cells, in the theta and gamma (40–80 Hz) frequency range, respectively (Pike et al., 2000).

Intrinsic properties render cells more sensitive to specific inputs according to their frequency content, which may contribute to the expression of different firing patterns as a function of the oscillatory context. Interestingly, the same concept applies to synaptic transmission, which can display remarkable frequency dependency.

## Underlying mechanisms: synaptic (short term plasticity)

Numerous studies have shown that synaptic transmission is not linear. The effects of an action potential on the postsynaptic neuron vary from one spike to the other, according to the history of the synapse, and its intrinsic presynaptic and postsynaptic properties. For example, synaptic transmission can display short term depression or facilitation in a frequency-dependent manner (Izhikevich et al., 2003). The combination of depressing and facilitating synapses also produces a pass band filter (Figure 2B). Such short term plasticity is influenced by the residual Ca^2+^ concentration in the presynaptic terminals, the activation of presynaptic metabotropic receptors, the desensitization of postsynaptic receptors etc. For example, excitatory postsynaptic currents in principal cells and GABA neurons display strong frequency-dependent depression via presynaptic mechanisms, whilst inhibitory postsynaptic currents generated by basket cells show less depression (Galarreta and Hestrin, 1998). Since excitatory and inhibitory pathways show different frequency sensitivity, the ratio between excitation and inhibition is also frequency-dependent (Varela et al., 1999). This means that some “hard-wired” neuronal connections will be functionally expressed according to the ongoing activity, creating functional neuronal assemblies and transiently linking local networks and networks of networks. One striking example is provided by the projection from glutamatergic dentate granule cells to CA3 pyramidal cells (Figure 2C). When activated at low frequency (10 Hz), dentate granule cells activate more GABA neurons than pyramidal cells, resulting in a strong inhibition of pyramidal cells. However, at higher frequency (40 Hz), GABAergic neurotransmission switches from short term potentiation to short term depression; the collapse of inhibition enabling the firing of pyramidal cells (Mori et al., 2004). A similar mechanism is involved in the switch between somatic to dendritic inhibition at 50 Hz (Pouille and Scanziani, 2004).

These examples show that the nature of the ongoing oscillations directly influences the functional connectivity, dynamically shaping the organization of functional local and large-scale networks in a behavioral relevant fashion. Hence, according to the oscillatory context, connections may be turned on or off, dynamically, changing the output pattern of individual cells, hence of the network.

The previous considerations show that firing patterns and functional connectivity can change dynamically in different oscillatory contexts. Since oscillations are emergent properties of networks, firing patterns, functional connectivity and oscillations are part of the same process, i.e., they determine/influence each other. How can then we explain the switch from one oscillatory mode to another? One possibility would be the intervention of external drivers that would change the functional state of circuits. Neuromodulators can fulfill such function.

## Underlying mechanisms: neuromodulators

Numerous modulators can be released by specific types of neurons. These neuromodulators include serotonin, acetylcholine, dopamine, and noradrenaline (Sara, 2009). Serotoninergic neurons of the Raphe control sleep-wake behavior (Monti, 2011). Their stimulation directly activates hippocampal GABA neurons, resulting in an inhibition of pyramidal cells (Varga et al., 2009).

Basal forebrain structures provide cholinergic inputs to numerous structures. Basal forebrain neurons are active during waking and quiescent during sleep. The release of acetylcholine can directly change the intrinsic properties of neurons and functional connectivity. Cholinergic activation increases membrane potential oscillations (Chapman and Lacaille, 1999) and spike reliability (Lawrence, 2008) during theta frequency oscillations, and changes the firing pattern of neurons via the activation of Ca^2+^-dependent K^+^ channels (Griguoli and Cherubini, 2012). Functional connectivity can also be affected as activation of presynaptic nicotinic receptors increases neurotransmitter release (Griguoli and Cherubini, 2012). Finally, specific neuron types can be turned on as acetylcholine depolarizes CCK−, but not parvalbumin-containing, basket cells (Lawrence, 2008).

Locus coeruleus neurons are the sole source of noradenaline in the brain. They play a key role in attention and memory processes. Activation of these neurons decreases spike jitter and fine tunes sensory responses. The release of noradenaline decreases the activity of Ca^2+^-dependent K^+^ channels (thus the firing pattern) and functional connectivity, increasing GABAergic inhibition, and enhancing or decreasing glutamatergic transmission in a behavior-dependent manner (Sara, 2009).

Hence, neuromodulators specifically released during different brain states (sleep-wake cycle, attention etc.) have the potential to dynamically change the intrinsic properties and the functional connectivity, hence information processing at the single cell and network level.

In conclusion, as assessed by their firing patterns, neurons perform brain-state dependent computation. The switch from one mode to another can be explained, in part, by a dynamical reconfiguration of their intrinsic properties and of the functional connectivity matrix that links them to other neurons. Neuromodulators can perform such reconfigurations. But other mechanisms, linked for example to energy metabolism or circadian rhythm (via epigenetic processes), are likely to be involved. All these examples demonstrate the versatility of neuronal networks, which are able to reconfigure themselves dynamically in a brain state-dependent manner, thus increasing the computational power of the brain.

### Conflict of interest statement

The authors declare that the research was conducted in the absence of any commercial or financial relationships that could be construed as a potential conflict of interest.
